# An Architecture Framework for Orchestrating Context-Aware IT Ecosystems: A Case Study for Quantitative Evaluation †

**DOI:** 10.3390/s18020562

**Published:** 2018-02-12

**Authors:** Soojin Park, Sungyong Park, Young B. Park

**Affiliations:** 1 Graduate School of Management of Technology, Sogang University, 35 Baekbeom-ro, Mapo-gu, Seoul 04107, Korea; 2 Department of Computer Science and Engineering, Sogang University, 35 Baekbeom-ro, Mapo-gu, Seoul 04107, Korea; parksy@sogang.ac.kr; 3 Department of Software Engineering, Dankook University, 152 Jukjeon-ro, Suji-gu, Yongin-si, Gyeonggi-do 16890, Korea, ybpark@dankook.ac.kr

**Keywords:** Internet of Things, IT ecosystem, cyber physical systems, self-adaptive systems, orchestration, MAKE-K (Monitor-Analyze-Plan-Execute over a shared Knowledge) loop

## Abstract

With the emergence of various forms of smart devices and new paradigms such as the Internet of Things (IoT) concept, the IT (Information Technology) service areas are expanding explosively compared to the provision of services by single systems. A new system operation concept that has emerged in accordance with such technical trends is the IT ecosystem. The IT ecosystem can be considered a special type of system of systems in which multiple systems with various degrees of autonomy achieve common goals while adapting to the given environment. The single systems that participate in the IT ecosystem adapt autonomously to the current situation based on collected data from sensors. Furthermore, to maintain the services supported by the whole IT ecosystem sustainably, the configuration of single systems that participate in the IT ecosystem also changes appropriately in accordance with the changed situation. In order to support the IT ecosystem, this paper proposes an architecture framework that supports dynamic configuration changes to achieve the goal of the whole IT ecosystem, while ensuring the autonomy of single systems through the collection of data from sensors so as to recognize the situational context of individual participating systems. For the feasibility evaluation of the proposed framework, a simulated example of an IT ecosystem for unmanned forest management was constructed, and the quantitative evaluation results are discussed in terms of the extent to which the proposed architecture framework can continuously provide sustainable services in response to diverse environmental context changes.

## 1. Introduction

We live in an age where almost every hour of our daily routine involves interactions with devices powered by software applications. Many of those devices are equipped with features that enable them to intake information and make autonomous decisions. The prominent emergence of new system operation paradigms, such as cloud computing or the Internet of Things (IoT) [1,2,3,4,5], implies that software products are rapidly moving away from the traditional concept of a closed, stand-alone system to one of a system of systems involving multiple, heterogeneous systems that collaborate with each other. 

“System of systems”, a term that has been used since the 1950s, refers to a system or collective of independent constituent systems that perform in collaboration to achieve a single common goal [6]. Although the term “system of systems” has been used for more than 50 years, there is still no widely accepted definition of its meaning, but rather it is regarded as a taxonomic group name [7]. An IT ecosystem is one of the systems that belongs to the newly emerging system of systems along with the IoT. An IT ecosystem [8,9,10] is a system operation concept which has been proposed to embrace the recent technical developments. The concept proposes a special form of system of systems in which multiple locally adaptive systems collaborate interactively. The name of the concept refers to its similarity with biological ecosystems where constituent lifeforms interactively evolve their shapes and behaviors to achieve a common goal: survival. The primary challenge of an IT ecosystem is to provide control mechanisms that ensure the group’s survival through effective evolution while ensuring the individual autonomy of its component system. In recent years, cases of implementing IT ecosystems have been introduced in various types of application domains [11,12,13,14,15], but these are only example cases that show the feasibility of the IT ecosystem concept; no empirical evaluation results have yet been provided. 

In our research, we adopted dynamic component reconfiguration methods used in self-adaptive software as the control mechanism for the collaborative evolution of an IT ecosystem. Noticeable academic developments in recent self-adaptive software studies [16,17,18] include efforts that are focused on applying the Monitor-Analyze-Plan-Execute plus Knowledge (MAPE-K) loop [19] to various domain applications and analyzing their results. MAPE-K is a reference architecture that governs a software feedback loop used for monitoring and analyzing internal and external environments to detect contextual changes, developing a reconfiguration plan when changes are deemed necessary, and executing the established plan. The MAPE-K architecture also includes information about the software’s components and the knowledge shared between those components. While MAPE-K is an effective architecture that is helpful in supporting the concept of self-adaptive software implementation, existing research studies are restrictive in their application of it to a single, stand-alone system’s internal components and their capabilities.

The framework proposed in this paper is based on the MAPE-K architecture, but it has three differentiations compared to existing study results based on the MAPE-K architecture as a basic prototype. These are described as follows:

- The first is an extension of the application scope of the MAPE-K architecture. Several typical frameworks [20,21,22] have been previously proposed to implement the MAPE-K loop, each with their own advantages and disadvantages, but their driving goals are limited to dynamic component reconfiguration in a single system. In contrast, the proposed framework includes mechanisms for both local adaption and global adaptation to ensure service sustainability of the system’s individual components while maintaining IT-ecosystem-wide collaborative performance.
- The second is a proposal for an orchestration mechanism that can minimize overheads due to the expansion of the IT ecosystem scale. Generally, the scale of an IT ecosystem that survives to achieve the common goal changes in the open IoT environment. The increase in the population of participating systems in the IT ecosystem can be a factor that can increase the time needed to find the optimal combination of device collaboration. The framework proposed in this paper aims to solve the scalability problem of IT ecosystems by applying a genetic algorithm, which is considered one of the most proven methods to solve the problem that calculates the global optimal solution.
- The third is a quantitative evaluated framework to provide a walk-through of a sample case study. Though we could not build an IT ecosystem in the real world, we have developed a sample IT ecosystem utilizing the proposed conceptual architecture to showcase its operational capabilities in simulated environments. Extending our previous work [23], we continue to explain our dynamic reconfiguration mechanism by providing a sample case study, which is an unmanned forest management system, into an IT ecosystem domain. Our research results are differentiated from the findings reported in previous work in that they apply the proposed mechanisms to an actual working IT ecosystem, thereby grounding our performance results in quantifiable test results and reinforcing the feasibility of our suggested architecture framework.

The rest of this paper is organized as follows: Section 2 presents a quick review of previous research studies on self-adaptive software. Section 3 describes the concepts of the reference architecture that was presented in our previous work. Section 4 briefly introduces the operating environment of the IT ecosystem application for the sample target domain, which is unmanned forest management. Section 5 explains the dynamic reconfiguration mechanism employed by the system components that comprise the concrete architecture utilizing the reference architecture described in Section 3. Section 6 specifies the quantitative analysis data resulting from a feasibility evaluation of the suggested architecture. Lastly, in Section 7, we present the conclusions of our study along with our plans for future research. 

## 2. Related Work

Rainbow [20], MUSIC [21], and DiVA [22] are noticeable study results about frameworks that support a self-adaptive system. These frameworks were all proposed to implement the MAPE-K loop. Each of the frameworks proposed its own mechanism for monitoring and analyzing the environment data detected by the runtime inside or outside the software system, establishing a plan to respond to a significant environmental change if detected, and then executing the established plan. 

The Rainbow framework contributed to the first case that proposed an infrastructure, systematically, which can be reusable by separating application logic and adaptation logic. Since then, various types of adaptive software frameworks have been proposed using the Rainbow framework as a prototype. Although the Rainbow framework’s reusable infrastructure, relatively low cost, and low effort make it advantageous to achieve self-adaptation, it has a limitation in that only action rules that are specific to predefined circumstance are operable. 

MUSIC proposes a solution that combines component-based development and service-oriented architecture (SOA) to respond to requirements that are changed dynamically in a mobile environment. It resolves adaptation issues by combining components that are most suitable to given circumstances after classifying all the required components according to the business logic, context awareness, and interests considered during the adaptation. Since MUSIC does not propose a model of an MAPE-K adaptation goal in the framework, the adaptation plan has to be updated or, in some cases, replaced manually, which is a limitation of this framework.

DiVA focuses on providing a methodology and framework to either manage the variability of a self-adaptive system or develop a self-adaptive system, rather than proposing architecture about the infrastructure of adaptive software. The architecture of DiVA is based on aspect-oriented programming (AOP), and it supports self-adaptation by adding dynamically required aspects as a form of a plug-in.

The results of the studies investigating the Rainbow, MUSIC, and DiVA frameworks have their own advantages and disadvantages. Besides the previously mentioned framework related studies, various case studies on application of self-adaptive control loop are introduced [24,25,26,27]. However, until now, most of the proposed frameworks and applications based on a self-adaptive mechanism provide answers for local adaptation to dynamically change the configuration of software components inside a single system. Our research builds on the work presented in previous studies to extend the application of the dynamic reconfiguration mechanism of self-adaptive software from single system to entire IT ecosystems by bridging the technical gap between the self-adaptive framework and the conceptual architecture of IT ecosystems.

## 3. A Reference Architecture for IT Ecosystems

**Figure 1:**
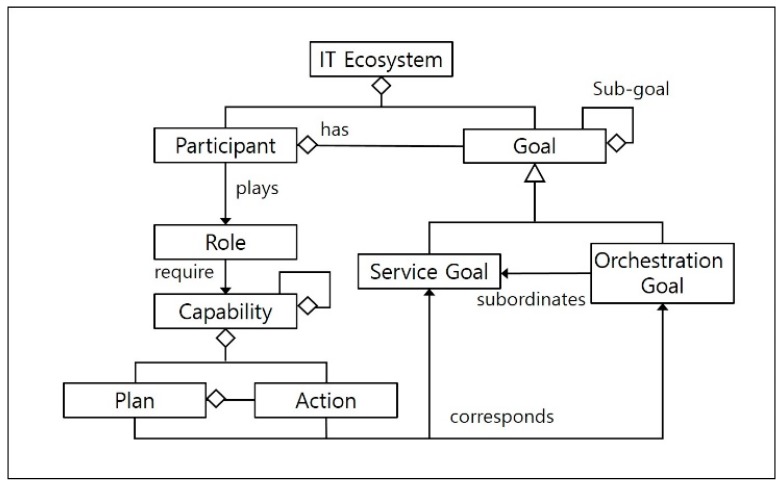
Metamodel for IT ecosystems [28]. Reproduced with permission from Soojin Park, Lee Seungmin, Young B. Park, A Reference Architecture Framework for Orchestration of Participants Systems in IT Ecosystems; published by Springer Nature, 2015.

In our previous research [28] we suggested a metamodel for defining IT ecosystems as a set of system goals and aggregate participant system collaborations based on Organization-based Multi-agent System Engineering (O-MaSE) [29]. In this paper, we extend that research by complementing the metamodel, as shown in Figure 1.

The new metamodel shown in Figure 1 presents the objectives of IT ecosystems as two separate goals: a service goal to specify the requirements of individual autonomous system participants in the ecosystem, and an orchestration goal to specify the operation performance and quality of the entire ecosystem. Each participant performs one or more roles with a predefined capability set for each role, in which a capability is defined as a set of actions that executes a specific plan.

Before developing reference architecture as a generic conceptual model to operate an IT ecosystem specified by the suggested metamodel in Figure 1, we need to define the two requirements that the reference architecture must satisfy. First, the reference architecture must specify the nature of the relationships between N instances of MAPE-K loops assigned to each participant system in order to achieve the service goal, in accordance with the single MAPE-K loop assigned to achieve the IT ecosystem’s orchestration goal. Second, the reference architecture must clarify the role that each participant system plays in order to achieve the two separate goal models, and it must specify the collaboration protocol that should be used. 

**Figure 2:**
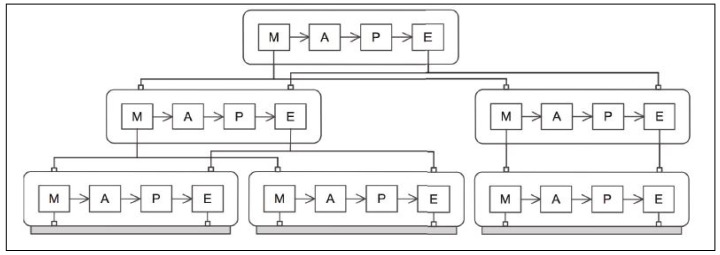
Hierarchical control pattern [30]. Reproduced with permission from Danny Weyns, Bradley Schmerl, Vincenzo Grassi et al., On Patterns for Decentralized Control in Self-Adaptive Systems; published by Springer Nature, 2013.

From among several multiple MAPE-K loop distribution patterns proposed in [30], we selected the hierarchical control pattern (Figure 2) as the base pattern of our suggested architecture framework. The reasoning behind this choice is that the hierarchical control pattern is the most efficient in the IT ecosystem context where multiple self-adaptive systems in distributed locations collaborate as constituents of a single ecosystem. As seen in Figure 2, this involves a pattern of N instances of MAPE-K loops in independent execution that are connected in a manner in which the modules within each MAPE-K loop that are responsible for monitoring or carrying out adaptation to locally detected environment changes share their local information or system capabilities with each other.

**Figure 3:**
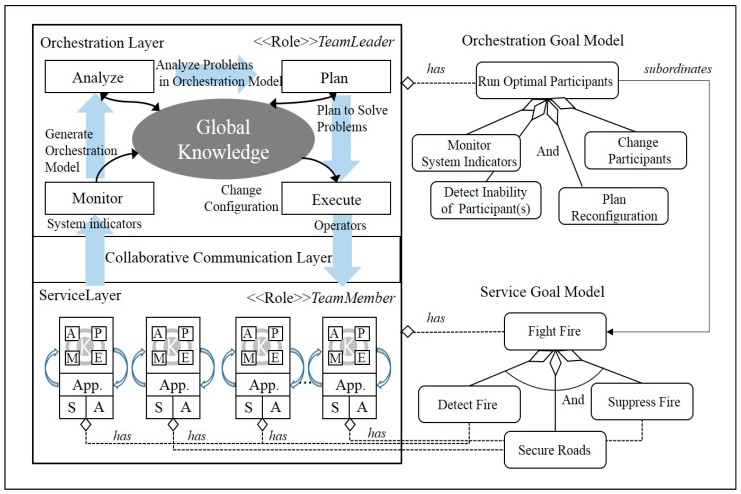
Conceptual architecture of an IT ecosystem [23]. Reproduced with permission from Soojin Park, Young B. Park, ITE arbitrator: A Reference Architecture Framework for Sustainable IT Ecosystems; published by IEEE, 2016.

Figure 3 presents a refined view of the conceptual reference architecture model for IT ecosystem operation based on the selected control pattern. Its overall structure suggests a vertical hierarchy composed of a single MAPE-K loop and N instances of MAPE-K loops, with respective purposes of achieving the ecosystem-wide orchestration goal and the component-level service goal. Each participant system defines its own metric for evaluating its service performance in correspondence to its given role, causing data to be periodically collected and stored according to the defined metrics within the local system. Among the locally stored knowledge, the information that needs to be shared globally is periodically updated to the global knowledge storage, enabling information sharing among the IT ecosystem participants. The sequence of knowledge sharing activities is carried out on the *Service* layer embedded in the systems designated to fulfil the *<<Role>> TeamMember*.

The MAPE-K loop running on the *Orchestration* layer continuously monitors a separate set of metrics defined to measure the status of the collaboration or the availability of the participating systems. When collaboration performance fails to exceed the IT ecosystem’s operation goals, the problem is analyzed in order to develop a plan to either disassociate the problematic participant from the collaboration or to introduce a new candidate system, and, thereafter, it is executed. The MAPE-K loop for satisfying the orchestration goal is executed by the *Orchestration* layer components embedded in the systems assigned with the *<<Role>> TeamLeader*.

The orchestration goal model and service goal model shown on the right-hand side of Figure 3 captures a snapshot of an IT ecosystem model for the unmanned forest management system that will be introduced in Section 4. The generic goals applicable to the entire IT ecosystem, such as “*Run Optimal Participants*”, are an example of a defined orchestration goal. Goals achieved as a result of functional collaboration of the participant-embedded capabilities, such as “*Fight Fire*”, are service goals. Subordinate goals are defined to achieve a higher-level goal, such as: “*Detect Fire*”, “*Secure Road*”, and “*Suppress Fire*”, are examples presented in Figure 3. How these subordinate goals are assigned to participants is determined by the participant’s capabilities. 

**Figure 4:**
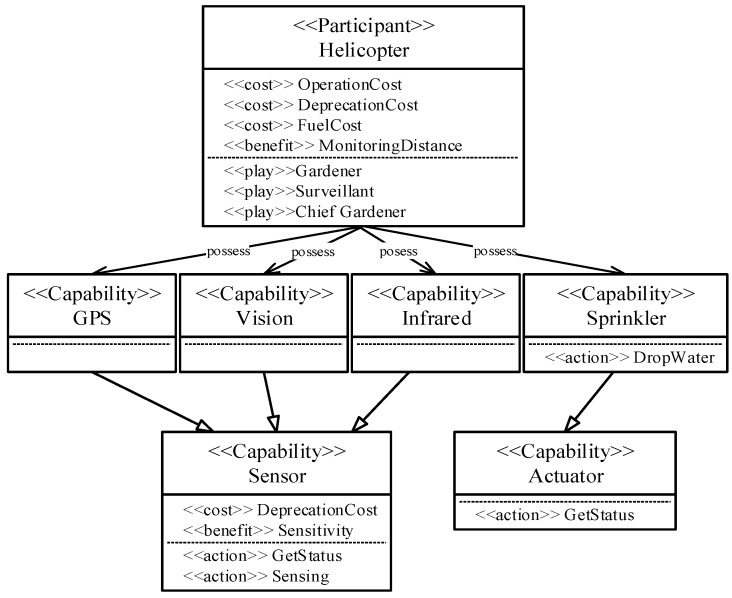
Model for a participant type: *Helicopter*.

For example, as seen in Figure 4, the unmanned helicopter type participant, defined using the metamodel shown in Figure 1, has a sensor and an actuator as its *<<Capability>>* element, and these both implement the <<action>> of *GetStatus* and *Sensing*. Based on these attributes, we can determine that the unmanned helicopter type participants are equipped with the necessary capabilities to achieve “*Detect Fire”*, which is a subordinate goal of the “*Fight Fire*” service goal.

The participant to be assigned the *<<Role>> TeamLeader* is selected from among all the IT ecosystem participants using a predefined strategy that defines the election policy, such as “*the system with the most expansive capabilities*” or “*the system with the highest current availability*”. The system designated as the *TeamLeader* searches for MAPE components within the *Orchestration* layer, binds them to its system, and thereby controls the configuration of the entire IT ecosystem.

**Figure 5:**
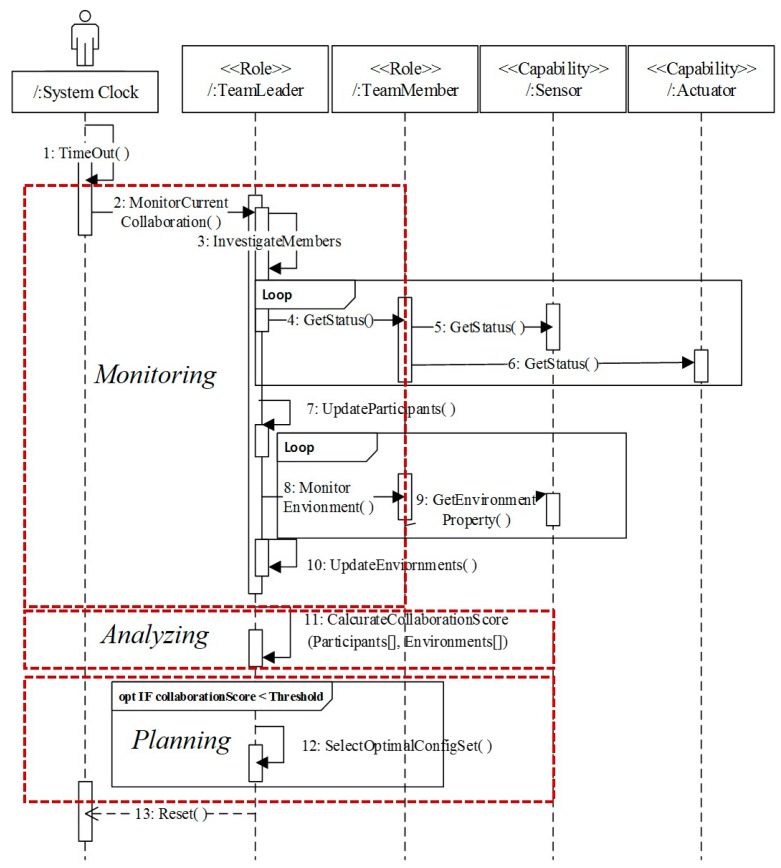
Definition of collaboration behavior between team leader and team member [28]. Reproduced with permission from Soojin Park, Lee Seungmin, Young B. Park, A Reference Architecture Framework for Orchestration of Participants Systems in IT Ecosystems; published by Springer Nature, 2015.

Figure 5 provides a definition of the collaboration protocol between the *TeamLeader* and *TeamMember* systems, triggered by an initialization of the *TeamLeader*, in order to achieve the orchestration goal “*Run Optimal Participants*”. The *TeamLeader* periodically monitors the status of its *TeamMember* systems by calculating the collaboration scores for each member using data collected from the sensors and actuators embedded in various *TeamMember* participants. When the collaboration score falls below a threshold, a reconfiguration of the optimal participants is carried out. Notice that the collaboration score is a type of utility function value that may vary widely with regard to the metric sets involved, depending on the orchestration goal that is to be achieved.

## 4. Case Study: An IT Ecosystem for Unmanned Forest Management

We continued to use the unmanned forest management in a simulated environment from our previous research [23] as the target domain of the application for our proposed architecture framework. The systems participating in the simulated ecosystem are 12 autonomous unmanned vehicles embedded with sensors and actuators, including unmanned aircrafts, unmanned helicopters, and unmanned vehicles.

**Figure 6:**
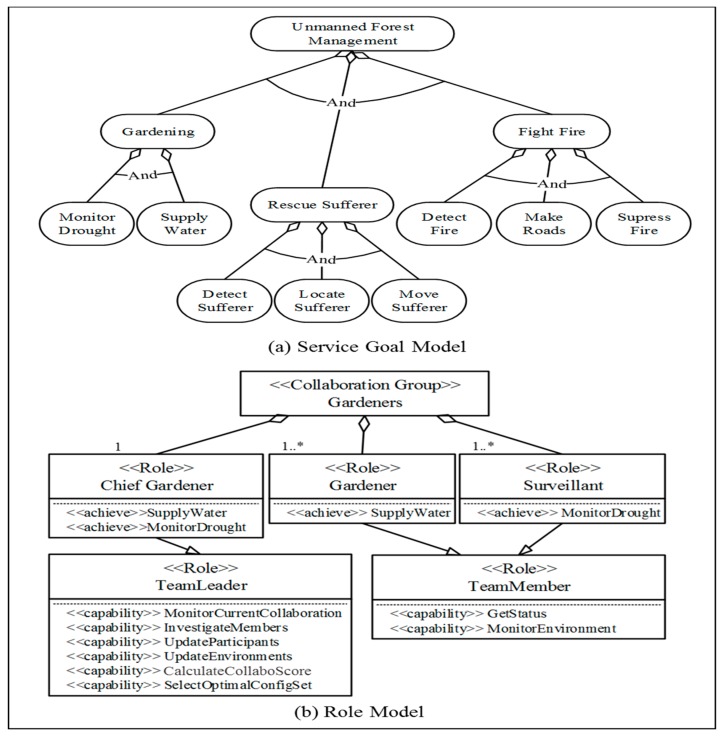
A service goal model and role model [23]. Reproduced with permission from Soojin Park, Young B. Park, ITE arbitrator: A Reference Architecture Framework for Sustainable IT Ecosystems; published by IEEE, 2016.

Figure 6a shows the full unmanned forest management IT ecosystem’s service goal model. Autonomous unmanned systems in the ecosystem perform either stand-alone or collaborative services, depending on the capabilities of each of the participants, such as rescuing survivors or planting trees. The *TeamLeader* role, which is defined in the collaboration behavior metamodel seen in Figure 5, is inherited by the *ChiefGardner* type, while the *TeamMember* role is inherited by the *Gardner* and *Surveillant* types, as seen in Figure 6b. The class diagram in Figure 6b describes how each instantiated role inherits *<<Capabilities>>* defined for the *TeamLeader* or *TeamMember* roles, and which goals each role will try to *<<achieve>>*.

**Figure 7:**
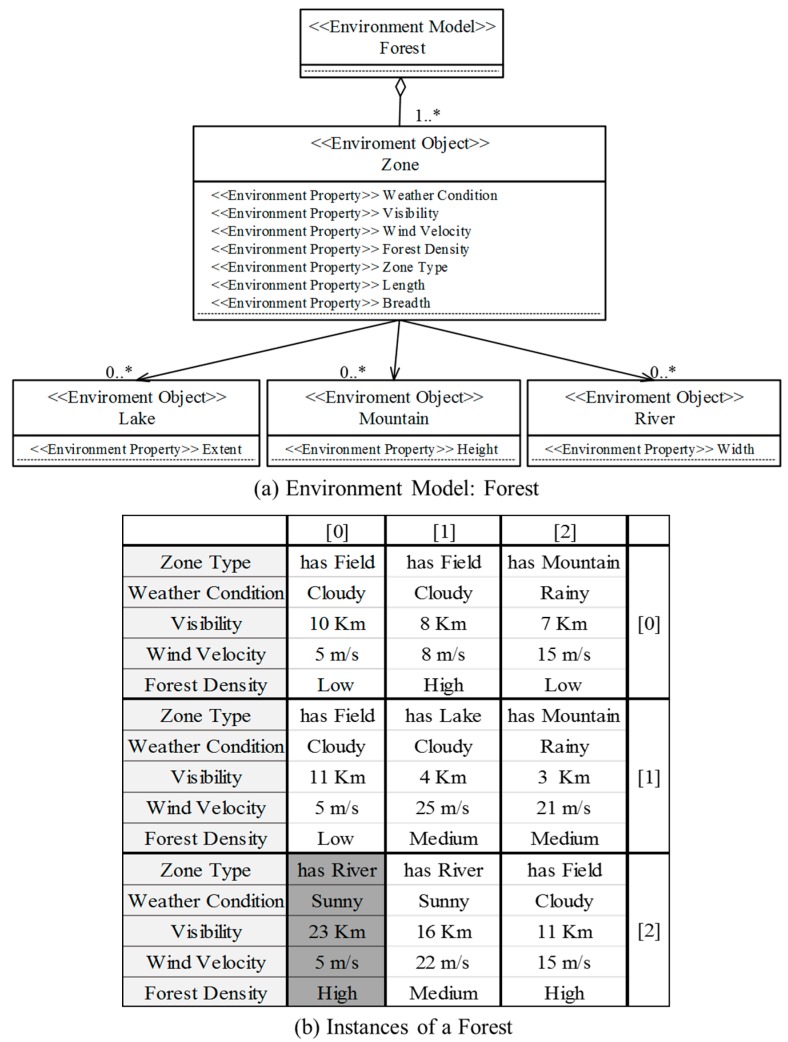
Environmental model for the unmanned forest management IT ecosystem.

Figure 7 shows the specification for the external environment in which the unmanned forest management IT ecosystem operates. The participant collaborations that occur across the nine forest regions are typically too vast for direct human management. Figure 7a depicts an environment model with a list of attributes that may manifest in the service zones. Each zone may have multiple categories of attributes; for example, weather attributes indicate a zone’s cloudiness, amount of rainfall, or fog density, while terrain attributes represent the zone’s visibility range, wind velocity, and forest density, and if bodies of water exist.

Figure 7b shows the status of the nine forest zones that are being monitored in terms of the defined attributes. In our simulated environment, the zones have identical size dimensions (width and breadth of 10 km), giving each zone an area of 100 km^2^. Therefore, we assigned zone width and breadth as the constants rather than the attributes to simplify the calculations. Changes in some of the zone properties, typically forest density or terrain type, are unlikely; thus, they are good constant candidates. However, they serve as constraints when determining unmanned system assignability. For example, zones with dense forests, rivers, or large lakes may prevent unmanned vehicles from being assigned to them or limit their utilization. Attributes that frequently change, such as weather conditions, visibility range, or wind velocity, may cause unmanned systems to be reassigned. For example, when dense fogs suddenly appear or visibility falls below a threshold, unmanned helicopter or aircraft operations may be suspended.

The cost and benefit metric items in collaborations between individual participant systems in the IT ecosystem vary depending on the service domain provided by the IT ecosystem. It is necessary to customize what cost items are required to run each of the participant systems in collaboration and what benefit items are provided as a result of collaboration in terms of the service viewpoints through the participant systems in the IT ecosystem to make them appropriate for each of the domain during the design of the IT ecosystem. For example, for JE1, which is a Jeep type, item J refers to an area of 0.64 ${\mathrm{km}}^{2}$ that can be monitored for one hour by JE1 in an environment where there is no mountain and no snow (rain) in the region. 

The values in Table 1 for the case study are inferred values obtained by reviewing the literature by vehicle or sensor type [31,32,33,34]. However, if all populations of the participants in the real IT ecosystem are prepared in the real world, the metric values for the cost–benefit evaluation can be calculated based on the premeasured historical data regardless of whether or not individual participant systems participate in the IT ecosystem collaboration. Other profiles of each unmanned system from a cost-benefit perspective is presented in Table 1. Information on the profile includes cost factors (columns A to G), representing the costs incurred by the system operations, and benefit factors (columns H to N), representing the performance of the sensors embedded in the system. Column O shows the remaining fuel and column P displays the available resources. The profile information is used as input data to calculate the collaboration scores, which is further described in Section 5.4.

## 5. An Instantiated Architecture for the Unmanned Forest Management IT Ecosystem

In this section, we look at the composition of a concrete architecture designed for unmanned forest management IT ecosystem operations, which is an instantiation of the proposed conceptual reference architecture discussed in Section 3. Furthermore, we explain the principles behind the self-adaptation mechanism of the architecture.

**Figure 8:**
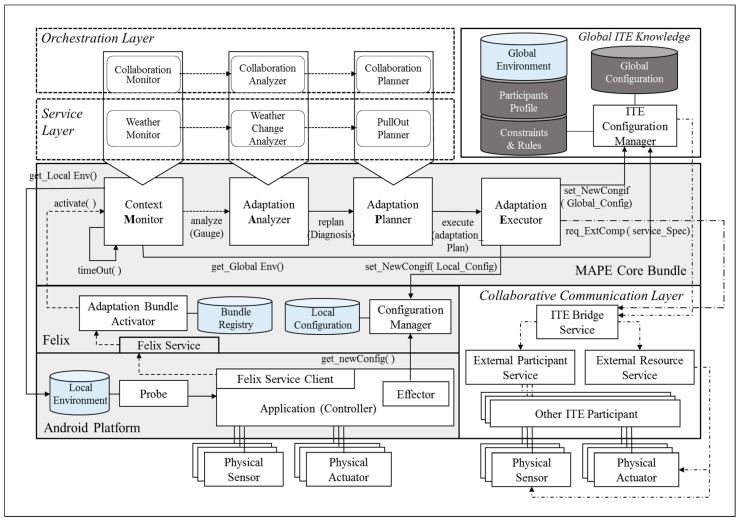
Concrete architecture for the unmanned forest management IT ecosystem.

Figure 8 shows the structure of a concrete architecture designed for the unmanned forest management IT ecosystem. All the participants in the ecosystem share the Android platform layer as their lowest layer; the Android platform layer and the Felix [35] layer are embedded in all systems by default, regardless of the system’s assigned role. To monitor the status of the applications that are running, we injected two special purpose components into the Android platform. The first is the *Probe* component, which is identical to the concept proposed in Rainbow [20], a widely used reference architecture that supports the MAPE-K loop. *Probe* acts as the channel for periodically receiving the attribute information needed to evaluate the status of the running applications, and it is capable of monitoring different metric sets depending on the target domain. For the unmanned forest management IT ecosystem, *Probe* uses metrics that read data from the system-embedded sensors, such as fuel gauges and GPS. The collected status data are first stored in the local environment storage within the system.

Another special purpose component injected into the Android platform layer is the *Effector*, which adds or removes components, such as sensors, actuators, or other system-internal components, based on the updated configuration information acquired from the *Configuration Manager* in the Felix layer.

Apache Felix is used as an interface between the Android platform and the *MAPE Core Bundle* layer. When the *Felix Service Client* initiates the *Adaptation Bundle Activator* using the Felix Service, the activator looks up the bundle registry information on the cloud and dynamically binds the necessary components to the *MAPE Core Bundle* layer.

Components bound to the MAPE Core Bundle layer are, thereafter, managed by a lifecycle management service provided by OSGi [36]. The role of the target system, to which the components are to be bound, determines the component sets that are used. For example, for systems assigned with the TeamLeader role Chief Gardner, the components defined in the Orchestration layer are bound to the MAPE Core Bundle layer to activate the MAPE cycle in order to achieve the orchestration goal. In contrast, for systems with TeamMemer roles Gardner or Surveillant, the components defined in the Service layer are bound to the MAPE Core Bundle layer. To better illustrate the integrated relationship between the local adaptation mechanism and the global adaptation mechanism (which we refer to as the orchestration mechanism), we will walk through a specific scenario where an unmanned aircraft in service is required to suspend its service due to a sudden change in its external environment. As we walk through the scenario, we will describe how the component roles in Figure 8 are performed.

### 5.1. Local Adaptation Mechanism for Achieving the Service Goals of Individual Participants

**Figure 9:**
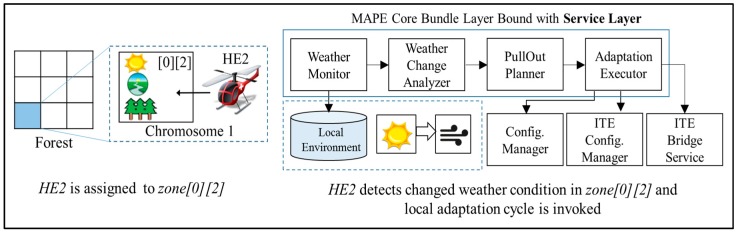
A sample scenario running the local adaptation mechanism.

Figure 9 depicts a situation where HE2, an unmanned aircraft carrying out the “*Monitoring Drought*” service goal, is met with a sudden gale of 25 m/s in *zone[0][2]*. The change of weather in the zone is detected by the sensor installed on HE2, and it is subsequently updated to its local environment storage. HE2’s assigned role *<<Surveillant>>*, which is a *TeamMember* type of role, triggers the components within the *MAPE Core Bundle* layer to be bound to the components in the *Service* layer. The *Service* layer may contain various component sets, depending on the service goal. In our scenario, the components in the *Service* layer (*WeatherMonitor*, *WeatherChangeAnalyzer*, and *PullOutPlanner*) are respectively bound to *ContextMonitor*, *AdaptationAnalyzer*, and *Adaptation Planner* in the *MAPE Core Bundle* layer. The MAPE-K loop triggered by the sudden weather change is executed among the HE2 components in the following order:

1. *Weather Monitor*: Periodically reads sensor data from the *Local Environment* storage, updates gauge values to reflect the current environment in *zone[0][2]*, and sends the data to *Weather Analyzer*.
2. *Weather Analyzer*: Analyzes the gauge values delivered from *Weather Monitor* to diagnose whether the present environmental conditions in *zone[0][2]* violate the HE2 assignment constraints, and requests a reconfiguration plan by sending the diagnostic results to *Pull Out Planner* as parameters.
3. *Pull Out Planner*: Creates a component reconfiguration plan for HE2 to land safely in a safe region in *zone[0][2]*, and it sends the plan to *Adaptation Executor*.
4. *Adaptation Executor*: If any component required by the reconfiguration plan does not exist in the given system, this component locates the missing component and identifies requests for it through *ITE Bridge Service* by passing the required service features as parameters.
5. *ITE Bridge Service*: This component is an OSGi bundle that provides a common interface for accessing the support devices available on the system; it also enables access to external resources. The REST [37] style services provided by *ITE Bridge Service* enable the entire IT ecosystem to share components, services, or other resources among all participants.
6. *Configuration Manager*: When all the components required to land HE2 have been procured, the component reconfiguration results are sent to *ConfigurationManager* within HE2, and updates its *Local Configuration* storage. Then, *Effector* in HE2’s controller reads the updated configuration and implements the actual component reconfiguration. Function calls between the Android platform and OSGi bundle are unidirectional to prevent *AdaptationExecutor* from directly passing on a new configuration to *Effector*.
7. *ITE Configuration Manager*: The changed environmental information in *zone[0][2]* and the status of HE2 (*incapable of service*) are updated to *ITE Global Knowledge* through *ITE Configuration Manager*.

### 5.2. Orchestration Mechanism for IT Ecosystem-Wide Dynamic Reconfiguration

**Figure 10:**
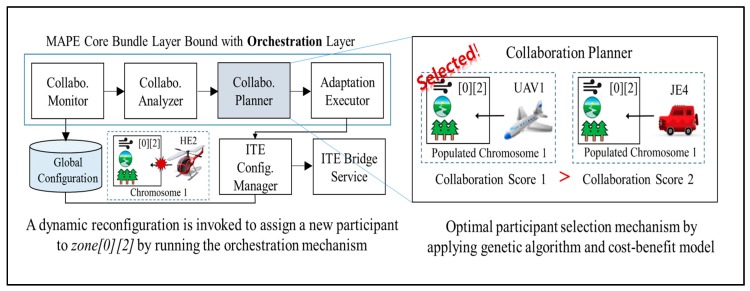
A sample scenario running the orchestration mechanism.

Column O in Table 1 shows that, in the unmanned forest management IT ecosystem, AP2 is selected for the *TeamLeader*-type role *ChiefGardner* because it has the highest availability (83%) among the candidate participants. As HE2 executes its local adaptation to land in response to sudden turbulence, the situation is updated to *ITE Global Knowledge*. As shown in Figure 10, the sequence of collaboration among the components bound to the *MAPE Core Bundle* layer within AP2 (the *TeamLeader*) is as follows:

- *Collaboration Monitor*: On every monitoring cycle, *Collaboration Monitor* acquires the current configuration information from the global configuration storage in *Global ITE Knowledge* and extracts the information needed to analyze the current collaboration score; it then delivers the information to *Collaboration Analyzer*.
- *Collaboration Analyzer*: This component receives monitoring information from *Collaboration Monitor* and calculates the collaboration score of the current configuration. Details of how the orchestration is measured as a score are provided in Section 5.4. If the score falls below a predefined threshold, *Collaboration Analyzer* reviews the mismatch between the current configuration and its environment, and it sends the result to *Collaboration Planner*.
- *Collaboration Planner*: This component receives information about the current configuration and its problems, and it plans the next optimal configuration. To do so, it calculates the scores for all possible configuration variations to identify the highest next score. When the next optimal configuration is found, *Collaboration Planner* develops a reconfiguration plan to execute the transition. These options include withdrawing an existing participant, deploying a new participant, or adding previously unavailable capabilities to the participant to activate unused sensors or actuators.
- *Adaptation Executor*: This component receives the reconfiguration plan developed by *Collaboration Planner*, and it selectively delivers commands that modify the *ChiefGardner* system’s local component configuration to *Configuration Manager* on the Felix layer. The delivered commands are updated to the local configuration. *Effector* on the Android platform periodically reads the updated commands and executes the component change. At the same time, reconfiguration commands intended for other *TeamMember* participants are delivered to their destinations through the *Collaborative Communication* layer. When the entire configuration of the IT ecosystem completes its shift to the next step, the changed configuration information is updated to *Global Configuration* storage via *ITE Configuration Manager*.

The quality of an IT ecosystem’s adaptation to the changes in its environment is influenced not only by the quality of its MAPE loop’s components, but also by the quality of the shared knowledge between systems. As shown in the scenario above, there are two types of knowledge storage in an IT ecosystem. *Global ITE Knowledge* stores the overall configuration of the IT ecosystem and the history of changes, and *Local Environment* storage manages the information needed to execute the local adaptations by individual systems. *Local Environment* storage is in the Android platform, while *Global ITE Knowledge* is on the cloud (for simplicity, Figure 8 represents the storage as being adjacently located). The data types stored in *Global ITE Knowledge* are:

- Environment model: This data type stores definitions for the environment components that change with time, shown in Figure 7a, and the environment specifications using the defined environmental model, as seen in Figure 7b. The environmental model does change after its initial definition at design, but the environment specifications are updated regularly based on the data collected through various sensors.
- Participants’ profile: This data type stores the profiles of individual participants in the IT ecosystem, including their operational costs and their expected benefits. The cost and benefit information is used as input to calculate the collaboration score when reconfigurations are needed.
- Constraints and rules: Information on the restrictions to be considered when assigning participants to specific zones is also stored in *Global ITE Knowledge* storage. In the case of the unmanned forest management IT ecosystem, the participants’ attributes determine the parts of the forest the system to which they can be assigned. For example, unmanned helicopters, unmanned aircrafts, or general aircrafts cannot be assigned to zones with a wind velocity higher than 20 m/s. *Collaboration Analyzer* monitors the list of environment-participant data pairs to detect violations of these constraints and rules. Table 2 shows a sample definition of the constraints for assigning unmanned systems performing the “Draught Monitoring” service goal.

### 5.3. Application of a Genetic Algorithm to the Participant Reallocation Problem in an IT Ecosystem

In the MAPE loop running in the *Orchestration* layer of a *TeamLeader* participant, *Collaboration Monitor* triggers a collaboration score recalculation once for every monitoring period in order to quantify the performance of the current collaboration. If the collaboration score falls below a predetermined threshold, *Collaboration Planner* calculates the collaboration scores of every possible configuration combination to find the next optimal value. Assuming an IT ecosystem has *n* participants and *m* service zones, the collaboration score calculation may be executed up to *nPm* times. Assuming the unmanned forest management IT ecosystem has 12 participants, one participant is assigned the *TeamLeader* role to manage the orchestration goal of the entire ecosystem, leaving 11 other candidate participants to be assigned to nine forest zones. This creates a total of 19,958,400 possible configurations (_11_P_9_) where each configuration requires a collaboration score calculation. Because collaboration score recalculation will be needed for every monitoring period, and IT ecosystem participants are likely to be embedded in systems that lack the processing power or computational resources of desktops or servers, the high overhead imposed by the collaboration score calculation can be prohibitive. Furthermore, an increase in the number of participants or service zones will increase the computational overhead exponentially. As a solution to the overhead problem, we applied a genetic algorithm to significantly decrease the number of configurations that require collaboration score recalculation.

While a genetic algorithm is a well-established approach for finding the global optimal solution for various decision making problems [38,39], it is not automatically applicable to all optimization problems. For a genetic algorithm to be applicable, two conditions must be met: (1) the optimal solution should be represented as a chromosome, or a sequence of bits (to enable the algorithm to generate solutions using transformative functions, such as crossover or mutations [40]), and (2) the optimal solution should be determined using a fitness function defined to evaluate the appropriateness of a given configuration-environment pair.

**Figure 11:**
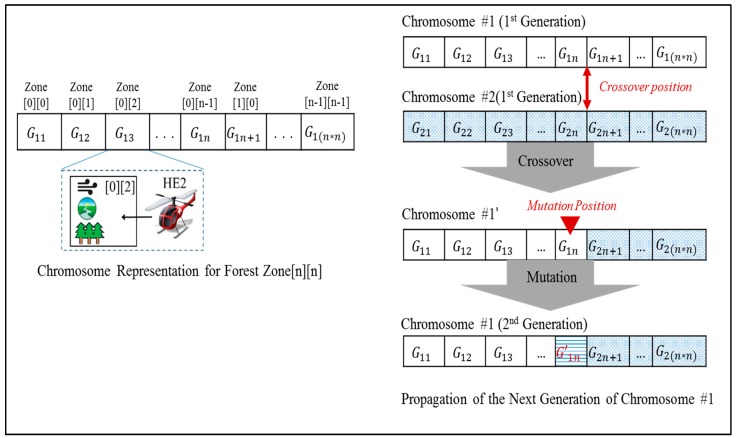
Propagating the next configuration of a UFM IT ecosystem by applying a genetic algorithm [23]. Reproduced with permission from Soojin Park, Young B. Park, ITE arbitrator: A Reference Architecture Framework for Sustainable IT Ecosystems; published by IEEE, 2016.

To apply a genetic algorithm to the problem of identifying the next optimal configuration, we have modeled the mapping between service zones and participant systems as a bit array, as shown in Figure 11. The attributes of individual forest zones and participants are encoded to represent the status of the collaboration at each monitoring interval as a form of genetic sequence.

*Collaboration Planner* creates the next generation of chromosomes by transforming them using simulated evolutions, such as genetic crossovers or mutations. After obtaining a set of candidate chromosomes, their genetic sequences are scanned to identify the genes (including data pairs) that violate configuration constraints (as seen in Table 2), which are consequentially excluded from the candidate pool. The remaining candidates are evaluated using the fitness function to determine the next optimal configuration. Each chromosome’s fitness for the unmanned forest management IT ecosystem is obtained using the collaboration score calculation function, which is explained in depth in the paragraph below.

The number of chromosomes needed to generate the next generation can vary depending on the problem that needs to be solved. In the worst case, when generating the very first configuration after initializing a new IT ecosystem, the number of required collaboration score calculations may be equal to the case in which a genetic algorithm has not been applied. However, once the first chromosome representing the initial optimal configuration has been selected, the collaboration score calculations for the following generations can be set to a fixed number; this is possible due to a genetic algorithm’s feature of fixing the size of the chromosome pool for the generations following the first selection of the optimal chromosome. This useful capability significantly contributes to reducing the computation overhead in dynamic reconfiguration, even when the number of participants or service zones increases. The impact of applying a genetic algorithm is further explored in Section 6, along with the results of the quantitative experiment.

### 5.4. Calculating the Collaboration Score via Cost–Benefit Analysis

In this section, we review the design of the fitness function, which is the second requirement for applying a genetic algorithm. We have defined a generic cost-benefit analysis model to measure the degree of collaboration between the unmanned systems participating in an IT ecosystem. The metrics for cost-benefit analysis may vary widely depending on the domain of the ecosystem; for unmanned forest management, we chose the metrics shown in Table 3. The factors that incur economically measurable costs as a result of participant operations are accounted for as cost factors. The metrics that reflect the degree of correctness and completeness of the collaboration are reflected as benefit factors. Different sets of metrics might need to be defined for other domains.

Cost factors include labor costs for operating the participating unmanned systems, depreciation costs for the unmanned system assets during their operation hours, fuel costs to run the systems, and depreciation costs for various sensor assets, including optical, vision, infrared, and GPS. Labor costs are accounted for because even self-adaptive systems need human intervention during emergencies, such as fighting a forest fire. Fully autonomous systems with a labor cost of zero indicate a system without any human intervention. Costs are evaluated per participant system, and then they are totaled to obtain the final cost.

The benefit derived from a given participant’s services can vary widely depending on the environment. This indicates that benefit factors are not determined by the participants themselves but by the mapping between the participants and their assigned service zones. While cost factors are deterministic when a participant is chosen, benefit factors are measurable only when they are assigned to a service zone and are in collaboration with that zone. Therefore, benefit factors are often used as an average of actual in-service values measured via prolonged monitoring. In case of the “Draught Monitoring” service goal, its benefit factors mainly focus on the performance of system-embedded sensors, including optical, vision, infrared, and GPS. In addition to the sensitivity and accuracy of the monitoring, the coverage of sensors, in terms of area per unit time (km^2^/h), is also accounted for as a benefit factor. Note that the monitoring coverage may vary in the same service zone depending on the weather condition.

Now, we explore the collaboration score calculation function in detail. The function requires an input of configuration data $Conf_{r}$, which is defined as a data pair $\left(p_{i},\;e_{j}\right)$ indicating an unmanned system $p_{i}$ assigned to a service zone $e_{j}$.

As described in Section 5.3, $cost\left(p_{i}\right)$ is solely determined by the choise of the participant $p_{i}$. The value of $cost\left(p_{i}\right)$ is calculated by adding up the normalized value $Cf_{k}$ of the pertinent cost factors listed in Table 3. For each cost factor, the weight coefficient $\eta_{k}$ representing each factor’s significance is applied; the sum of the weights should add up to 1:

(1) $$Ncf_{k}=\;\frac{\left(Cf_{k}-\;Cf_{min}\right)}{\left(Cf_{max}-\;Cf_{min}\right)}\;\mathrm{where}\;k=1,2,\ldots ,\;n$$

(2) $$cost\left(p_{i}\right)=\;\underset{k}{\sum }\left(\eta_{k}\times Ncf_{k}\right)\;\mathrm{where}\;\underset{k}{\sum }\eta_{k}=1$$

The expected benefit of assigning a participant $p_{i}$ at zone $e_{j}$, expressed as $benefit\left(p_{i},\;e_{j}\right)$, is calculated by adding up the normalized $Mbf_{k}$ values of relevant benefit factors weighted with $\eta_{k}:$

(3) $$Mbf_{k}=\;\frac{\left(Bf_{k}-\;Bf_{min}\right)}{\left(Bf_{max}-\;Bf_{min}\right)}\;\mathrm{where}\;k=1,2,\ldots ,\;m\;$$

(4) $$benefit\left(p_{i},\;e_{j}\right)=\;\underset{k}{\sum }\left(\eta_{k}\times Mbf_{k}\right)\;\mathrm{where}\;\underset{k}{\sum }\eta_{k}=1$$

The collaboration score is calculated using the $cost\left(p_{i}\right)$ and $benefit\left(p_{i},\;e_{j}\right)$ values obtained using Equations (1)–(4), presented above, and it can be adjusted by using appropriate weights when there are significant differences between the importance of the costs and benefits. If there are no significant differences, the delta between the cost and benefit is used as the collaboration score, as shown in Equation (5), promoting maximized return-on-investment. The total collaboration score for a configuration $Conf_{r}$ is equal to the sum of the collaboration scores for all data pairs $\left(p_{i},\;e_{j}\right)$ in the configuration $Conf_{r}$:

(5) $$CollaborationScore\left(p_{i},\;e_{j}\right)=\;w_{0}\;\times benefit\left(p_{i},\;e_{j}\right)+w_{1}\;\times \;cost\left(p_{i},\;e_{j}\right)\;\mathrm{where}\;\sum^{\text{​}}\left|\omega_{i}\right|=1\;\mathrm{and}\;\prod^{\text{​}}\omega_{i}=\;-1$$

Equations (6)–(8) show the steps used to calculate the collaboration score, assuming *UAV1* has been assigned to *zone[0][2]* (as seen in Figure 9). The operating cost for *UAV1* in executing the *Draught Monitoring* service goal consists of depreciation costs for the system asset and sensor assets, and fuel cost. Each cost factor is assigned an equal weight of 1/3. Regarding the benefit factor weights, sensor sensitivity (an accuracy metric) is assigned less weight than monitoring coverage (a collaboration quality metric), and each is given as 0.3 and 0.7, respectively. The following equations are the results of applying the *UAV1* profile data from Table 1:

(6) $$Cost\left(UAV1\right)=\frac{1}{3}*DeprecaionCost\left(UAV1\right)+\frac{1}{3}*FuelCost\left(UAV1\right)+\frac{1}{3}*DeprecationCostof\;Sensor\left(UAV1\right)=\frac{1}{3}*N\left(100\right)+\frac{1}{3}*N\left(70\right)+\frac{1}{3}*\left(N\left(86\right)+N\left(50\right)+N\left(30\right)\right)=0.31$$

(7) Benefit(UAV1,zone[0][2])=0.3*Sensitivity of Sensors(UAV1,zone[0][2])+ 0.7*MonitoringCoverage (UAV1, zone[0][2])=0.3*(N(0.92)+N(1)+N(1))+0.7*N(100)≈1.02

where N(x) is a normalized function for each factor:

(8)  CollaborationScore(UAV1, zone[0][2])=Benefit(UAV1,zone[0][2])−Cost(UAV1, zone[0][2]) =0.71

The remaining eight data pairs in $Conf_{r}$ are sequentially processed to calculate their collaboration scores by repeating the above steps. The added total for each candidate configuration is then compared to select the configuration with the highest sum, which will be the next optimal configuration.

**Figure 12:**
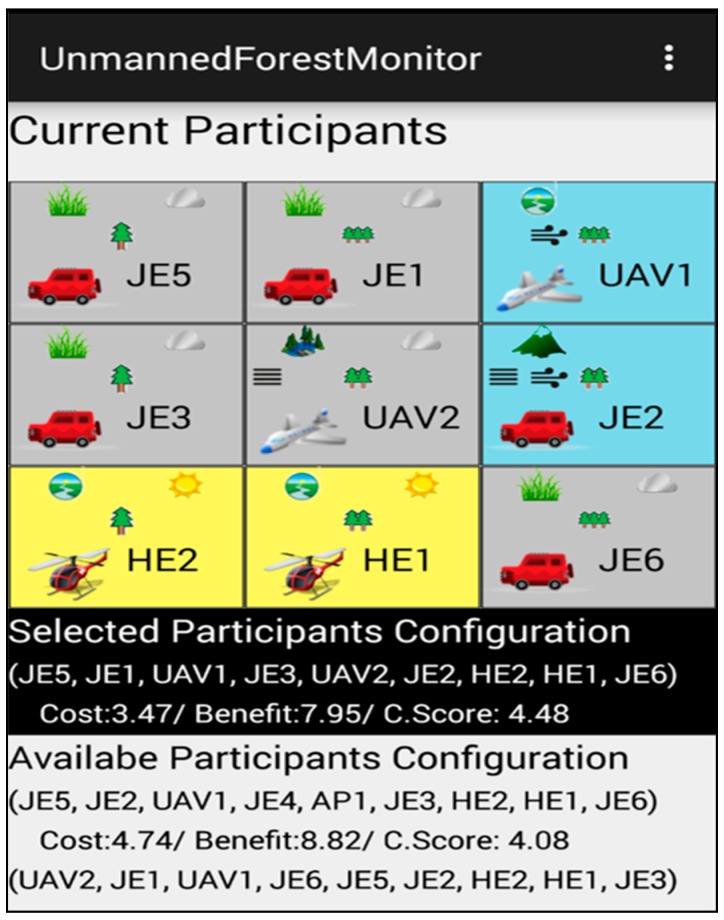
A configuration displayed on the screen of a mobile device [23]. Reproduced with permission from Soojin Park, Young B. Park, ITE arbitrator: A Reference Architecture Framework for Sustainable IT Ecosystems; published by IEEE, 2016.

Figure 12 illustrates the collaboration results displayed on a mobile device running an Android platform. We can observe the visualization of the currently selected configuration and its total collaboration score of 4.48. In addition to the selected optimal configuration, information on other candidate configurations is also shown at the bottom of the screen. 

## 6. Evaluation

This section discusses the quantitatively validated results that contribute to the IT ecosystem via the architecture framework that supports the dynamic orchestration proposed in this study. Toward this end, two questions will be validated:

1. Can the proposed framework guarantee that constant services will be provided while adapting to the internal and external environments faced by the overall IT ecosystem? (sustainability issue)
2. Is the overhead required for reconfiguration of the IT ecosystem an acceptable level, and does it not show an exponentially increasing pattern as the IT ecosystem expands? (scalability issue)

### 6.1. Design of Experiment

#### 6.1.1. Evaluation Scope 

**Figure 13:**
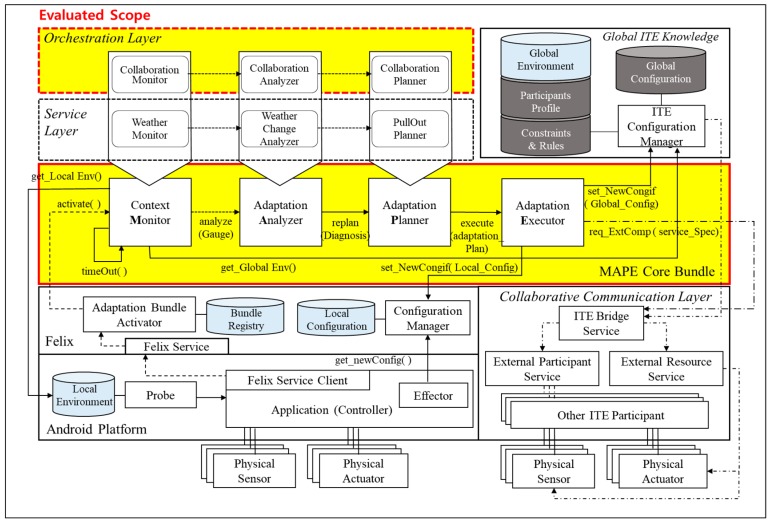
Scope of evaluation.

There are many current limitations to implementing an unmanned forest management system in the real world that can evaluate the overall performance of the proposed architecture framework specified in Figure 8 in Section 5. The most critical part among the components in the framework proposed in this paper is the *Orchestration* layer that recognizes environmental changes and modifies the optimal system collaboration configuration dynamically to maintain the sustainability of the IT ecosystem. Thus, this study set the components in the *Orchestration* layer, which was bound to the stub component over the *MAPE Core Bundle* layer inside the *TeamLeader* participant system, as a practical performance evaluation scope of the orchestration mechanism while the simulated unmanned forest management system was running. To help with understanding, the evaluation scope is highlighted among the overall structure of the proposed architecture framework in Figure 13.

#### 6.1.2. Metrics for Evaluation 

To test the sustainability and scalability issues that were set as the goal of the evaluation, the following metric sets are placed:

1 Periodically monitored collaboration score value

The performance of the orchestration mechanism that is conducted in the *MAPE Core Bundle* layer by the components that belong to the *Orchestration* layer was evaluated using the collaboration score, which is a result value of the fitness function presented in Section 5.4. A trend of the change in collaboration scores measured after executing the orchestration mechanism in response to various external environment changes is analyzed and discussed in the description of the evaluation results.

2 The mean required time from sensing an external environmental change by the *Collaboration Monitor* that belongs to the *Orchestration* layer via the *Collaboration Analyzer* up to the creation of the next optimal configuration plan by the *Collaboration Planner*

There are various practical overheads and delay factors, such as a delay in the collaborative communication layer or a delay in the sub-layer of the system, to provide services after a new participant system is positioned in a specified location or when participant systems in collaboration are disabled or new functions are redeployed during the execution of dynamic reconfiguration in the IT ecosystem that is running in the real world. However, since only overhead elements that were measurable in the simulated environment are considered currently, the overhead elements were not included in the current evaluation result values. A mean required time to create the optimal configuration plan was calculated based on 30 dynamic reconfiguration executions.

#### 6.1.3. Test Bed Specification 

We have conducted our experiments with a simulation of the unmanned forest management IT ecosystem implemented in Java and run on a computer with an Intel i7 processor 3.5 GHz and 16 GB of RAM.

### 6.2. Experimental Results

The verification result of whether or not the IT ecosystem is provided constantly, which answers the first question, is as follows. A ceaseless change in weather plays a role as an external factor that causes the evolution in the IT ecosystem of the unmanned forest management system, although the result is based on a simulated environment. Simulations were conducted to progress the wind intensity, rain travel path, and locations of fog and clouds across nine forest zones at a certain rate and direction for weather change. We aimed to prove the adaptation effect of the proposed mechanism by comparing the cost, benefit, and collaboration score for each of the IT ecosystem configurations measured for cases in which the orchestration mechanism in response to the environment change was or was not applied. Furthermore, this study aimed to verify if the proposed orchestration mechanism could provide a constant performance of collaboration services for the IT ecosystem, even in rapid environmental changes, by simulating a differential degree of weather changes.

In addition to changes in the external environmental factors, the unmanned forest management IT ecosystem has an internal factor, fuel consumption, which causes a service outage in the participating systems. Simulation was set to decrease fuel consumption at a certain rate according to the type of unmanned system over time by defining fuel consumption per hour. Thus, a system service outage due to fuel depletion can be another factor of the dynamic reconfiguration mechanism.

**Figure 14:**
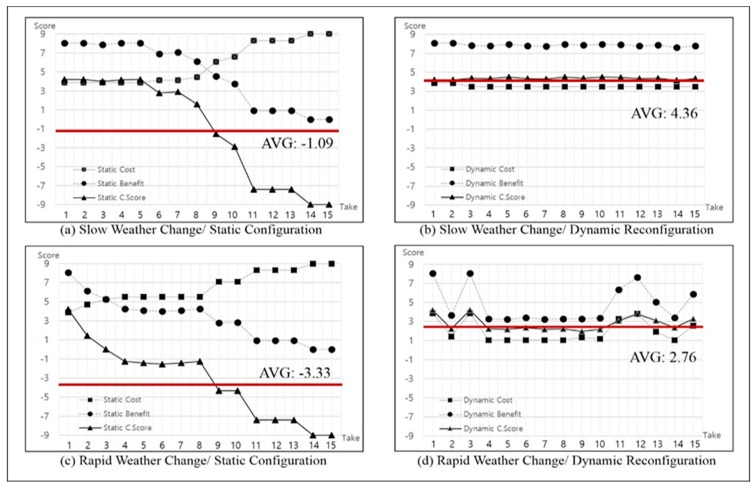
Calculated cost–benefit values and collaboration score for the selected optimal configuration extracted from each cycle: (**a**,**c**) without the proposed framework vs. (**b**,**d**) with the proposed framework.

The graphs presented in Figure 14 show a trend of collaboration performance changes in the IT ecosystem for the unmanned forest management system calculated by the *Collaboration Analyzer* for every monitoring cycle. Graphs (a) and (c), on the left side of Figure 14, indicate the collaboration performance monitoring results when the MAPE loop is not running at the *Orchestration* layer of the proposed framework, and graphs (b) and (d), on the right side of Figure 14, show the results when the MAPE loop is running. The horizontal axis in the graph represents the monitoring cycle that is performed. The vertical axis represents a cost value, benefit value, and collaboration score for the collaboration configuration at the monitoring cycle. The suspension of all the unmanned forest management system services assigned to nine zones was verified at the 15th cycle monitoring time when the orchestration mechanism is not running, regardless of the severity level of the weather change. Consequently, after the 15th cycle, the monitoring results are insignificant, which is why those results are not reflected in the graph.

As seen in Figure 14a, when the orchestration mechanism is not running, and the gradual weather change is simulated externally, a zone, where the participating system does not achieve the service goal, gradually occurs from the 6th cycle monitoring time. Thus, the benefit values and the collaboration scores start to decrease, and the values are rapidly dropped from the 8th cycle monitoring time. Conclusively, in the unmanned forest management system, nearly all the service regions become an outage state at the 14th cycle monitoring time, resulting in convergence of the benefit value to zero. As seen in Figure 14b, when rapid weather changes were simulated without running the orchestration mechanism, the service outage state in the unmanned forest management system started to occur from the 2nd cycle monitoring time, which was much faster than the service outage state in the gradual weather change scenario. Although the complete service age of the IT ecosystem was revealed at the 14th cycle monitoring time, which is the same in the above, the mean collaboration calculation score was −3.3, which was much lower than −1.09 at the time when a gradual weather change occurred. As expected, the decrease in the collaboration performance rate was much greater when the level of external change was rapid.

The two graphs on the right side of Figure 14 show the results that were obtained when the dynamic reconfiguration mechanism provided by the proposed framework revealed a completely different pattern. The measurements in Figure 14a, where the reconfiguration mechanism is not running, exhibit a descending curve from the 6th cycle monitoring time. In Figure 14b which depicts the results when the dynamic reconfiguration mechanism is running, the measurement maintains constant values for the cost, benefit, and collaboration score based on when the IT ecosystem operates until the 15th cycle monitoring time. On the lower side of Figure 14, graphs (c) and (d) display a comparison of the measurements when a rapid external change occurs, and the result shows the clear effect of the dynamic reconfiguration mechanism on maintaining constant performance services in the IT ecosystem. Thus, these verification results prove that the proposed orchestration framework contributes to maintaining the IT ecosystem via dynamic reconfiguration because the IT ecosystem appropriately responds to internal and external environmental changes.

Next, the second verification point sought to verify whether or not the overhead, due to the dynamic reconfiguration mechanism run by the MAPE-K loop that is implemented in the *Orchestration* layer, is acceptable. When the “*Drought Monitoring*” service was provided at nine zones by assigning 12 unmanned systems in the IT ecosystem for the unmanned forest management system discussed in this study, the average time it took to select the optimal configuration combination measured prior to applying a genetic algorithm was approximately 4.24 s. In contrast, when the optimal candidates were selected using the calculation of the collaboration score by generating a set of candidate configurations through the execution of a genetic algorithm via single thread, the measured average time at the same condition was only 2.77 milliseconds. This result can be regarded as the effect obtained by limiting the size of the next generation set generated by applying the genetic algorithm to 200. 

Since the time required to select the optimal configuration combination for the same circumstance was verified, this study aimed to verify which pattern would be expected by the overhead increment when the IT ecosystem range was expanded. Without applying the genetic algorithm, when the number of collaboration participation candidate systems was increased by one to 13 systems, the time required to select the optimal configuration measured at a desktop personal computer (PC) with a basic specification was approximately 14 min, on average, which was 800-times faster than the time required when using 12 systems. Once again, when an additional system was added for a total of 14 systems, it was not possible to obtain the results. 

**Figure 15:**
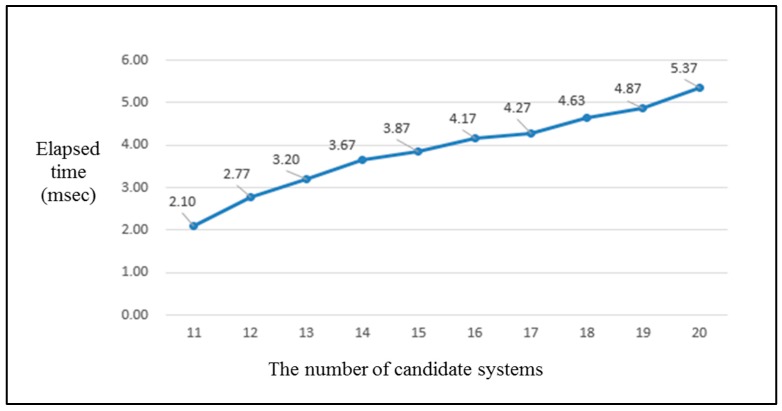
The trend of elapsed time required until the decision of the optimal configuration set from second reconfiguration for an increasing number of candidate systems.

In contrast, when the genetic algorithm was applied, the time required to select the optimal configuration measured while increasing the number of candidate systems that can participate in collaboration with the IT ecosystem from 11 to 20 candidates was increased from 2.10 milliseconds to 5.37 milliseconds, as shown in the graph presented in Figure 15. The increase in the pattern of time required to select the optimal configuration would be represented by a slope nearly close to a horizontal straight line if the unit of the vertical y-axis is a second instead of a millisecond. The trend increased because the time required to select the first optimal configuration increased in proportion to the increase in the number of participation systems, even though the size of the candidate set of the next optimal configuration was always limited to a constant number (although that number was minimal). However, the exponential increase in the time required to select the optimal configuration, which was discovered in the results of the previous experiment without applying the genetic algorithm, did not occur. The above verification result proves that the framework proposed in this study not only supported the consistency of the IT ecosystem, it also solved the scalability issue in the IT ecosystem although the result was measured based on a limited experiment result in the simulation environment.

## 7. Conclusions and Future Work

This study proposes a framework that supports the dynamic orchestration mechanism, which is one of the core requirements of an IT ecosystem, to ensure that it provides consistent services. The study complemented and individualized the metamodels that have been defined in previous studies to develop a framework that supports an IT ecosystem, which was tested using an unmanned forest management service system in an Android platform-based simulation environment. The proposed framework provides the orchestration mechanism by which environmental changes are recognized through constant monitoring. An optimal system collaboration configuration was selected for the changing environment because the IT ecosystem adapts and evolves according to environmental changes in a way that is similar to a biological ecosystem. The proposed framework uses a method that selects a candidate set of optimal configurations by applying a genetic algorithm to resolve the overhead issue due to the IT ecosystem expansion. As an evaluation function to select the optimal configuration from among the selected configuration candidates, the performance of collaboration between unmanned devices was quantified with cost and benefit factors, thereby defining a function that derives the collaboration score. 

Although previous adaptive system-related study results have proposed various types of adaptive software frameworks and infrastructures, they only focused on the self-adaptive technique of the configuration components inside a single system. In addition to studies investigating IT ecosystems, studies on the system of systems based on the IoT have also been conducted. However, most of those studies have only proposed conceptual architecture rather than practical, implementable case results. 

Thus, the present study is differentiated from previous studies in that it proves the effectiveness of the proposed framework using quantitative verification results that are applied to practical service examples by which an actual operation can be verified, even though it was conducted under simulated environments. The quantitative verification results were analyzed, and the results verified that the proposed framework was able to reassign a new configuration in accordance with the internal and external changes that occurred frequently in the IT ecosystem, thereby maintaining constant service. Furthermore, the experiment results proved that the problem of exponential increase in the overhead of dynamic reconfiguration, due to IT ecosystem expansion, can be resolved by limiting the size of the set of candidate configurations by utilizing a genetic algorithm. 

Up to now, our studies have focused on problem solving that selects optimal ecosystem participants to sustain the IT ecosystem. In a future study, the vulnerabilities inherent in the combination of already-selected optimal systems will be identified prior to collaboration execution. Toward this end, the potential conflict factors between the participating systems, which can act as vulnerabilities during collaboration, will be identified using formal model-based verification.

## Figures and Tables

**Table 1:** Participant profiles in the unmanned forest management IT ecosystem.

ID	Type	Cost						Benefit							O	P
		A	B	D	E	F	G	H	I	J	K	L	M	N		
JE1	Jeep	40	40	14	60	32	19	0.7	0.6	0.64	100	87	74	46	30	80
JE2	Jeep	40	40	15	35	37	22	0.59	0.35	0.73	100	78	54	54	25	69
JE3	Jeep	40	40	12	64	39	23	0.98	0.64	0.78	100	96	57	47	27	63
JE4	Jeep	40	40	13	76	35	21	1	0.76	0.7	100	86	58	46	37	79
JE5	Jeep	40	40	16	45	38	23	0.8	0.45	0.75	100	78	68	38	25	74
JE6	Jeep	40	40	16	85	47	28	0.93	0.85	0.93	100	84	72	51	31	84
UAV1	UAV	0	100	70	86	50	30	0.9	0.86	1	100	100	100	100	102	72
UAV2	UAV	0	100	75	100	50	30	0.95	1	1	100	100	100	100	86	68
HE1	Hlct	80	70	67	75	34	20	0.67	0.75	0.68	100	87	100	74	120	62
HE2	Hlct	80	70	56	60	43	26	0.87	0.6	0.85	100	87	100	78	116	83
AP1	AP	100	80	80	63	37	22	0.62	0.63	0.74	100	87	100	86	170	73
AP2	AP	100	80	83	73	47	28	0.84	0.73	0.93	100	83	100	87	150	86

**Table 2:** Constraints in the unmanned forest management IT ecosystem.

No.	Constraint for Assignment of Participant Systems
1	**IF** (*Zone[ ][ ].Weather.Visibility < 5 (km))* **THEN** *Helicopter* **CANNOT PLAY AS** *Surveillant* **AT** *Zone[ ][ ]*
2	**IF** (*Zone[ ][ ].Wind.Velocity > 20 (m/s)*) **THEN** (*UAV & Helicopter & AirPlane*) **CANNOT PLAY AS** *Surveillant* **AT** *Zone[ ][ ]*
3	**IF** (*Zone[ ][ ].Weather.Condition = Snowy*) **THEN** *Jeep* **CANNOT PLAY AS** *Surveillant* **AT** *Zone[ ][ ]*
4	**IF** (*Zone[ ][ ].Type = HasLake*) **OR** (*Zone[ ][ ].Type = HasRiver*) **THEN** *Jeep* **CANNOT PLAY AS** *Surveillant* **AT** *Zone[ ][ ]*
5	**IF** (*Zone[ ][ ].ForestDensity = High*) **AND** (*Zone[ ][ ]. Participant)= Jeep*) **THEN** *Jeep should be operated by a human driver*
6	**IF** (*Partcipant.RemainedFuelQuantity < Required Quantity for Running One Period*) **THEN** *Participant* **CANNOT PLAY** *any role* **AT** *every zone*

**Table 3:** Cost–benefit factors [23]. Reproduced with permission from Soojin Park, Young B. Park, ITE arbitrator: A Reference Architecture Framework for Sustainable IT Ecosystems; published by IEEE, 2016.

**Cost Factors**	
A	Cost of human labor for emergency operations ($/h)
B	Cost of system asset deprecation ($/h)
C	Cost of fuel ($/h)
D~F	Cost of sensor asset deprecation ($/h): vision (D), infrared (E), and GPS (F)
**Benefit Factors**	
G~I	Sensor asset sensitivity and accuracy (0~1): vision (G), infrared (H), and GPS (I)
J~M	Monitoring coverage (${\mathrm{km}}^{2}$/h): may vary depending on zone types or weather conditions J: Monitoring scope when there are no mountains and no snow (rain) in the region (${\mathrm{km}}^{2}$/h) K: Monitoring scope when there are no mountains and there is snow (rain) in the region (${\mathrm{km}}^{2}$/h) L: Monitoring scope when there are mountains and there is no snow (rain) in the region (${\mathrm{km}}^{2}$/h) M: Monitoring scope when there are mountains and snow (rain) in the region (${\mathrm{km}}^{2}$/h)
